# Tick fever risk and factors influencing the transmission of *Anaplasma marginale*, *Babesia bovis* and *Babesia bigemina* in cattle properties from Atlantic Forest biome

**DOI:** 10.1007/s11250-026-04866-5

**Published:** 2026-01-30

**Authors:** Juan Dario Puentes, Rosangela Zacarías Machado, Wendell Marcelo de Souza Perinotto, Vinícius Longo Ribeiro Vilela, Franklin Riet-Correa

**Affiliations:** 1 Programa de Pós-graduação em Ciência Animal nos Trópicos (PPGCAT), Universidade Federal da Bahia (UFBA), Av. Milton Santos, 500, Ondina, Salvador, BA 40170-110 Brazil; 2 Centro de Ciências Agrárias, Ambientais E Biológicas (CCAAB), Universidade Federal Do Recôncavo da Bahia (UFRB), Rua Rui Barbosa, 710, Centro, Cruz das Almas, BA 44380-000 Brazil; 3 Vector-Borne Bioagents Laboratory (VBBL), Department of Pathology, Reproduction and One Health, School of Agricultural and Veterinary Sciences (FCAV), São Paulo State University (UNESP), Jaboticabal, SP 14884-900 Brazil; 4 Imunodot Diagnósticos Veterinários, Rua Dr. Mario de Campos, nº1150, Jardim São Marcos I, Jaboticabal, SP 14887-200 Brazil; 5 Programa de Pós-Graduação em Ciência e Saúde Animal, Universidade Federal de Campina Grande (UFCG), Avenida Universitária s/n, Patos, PB 58708-110 Brazil; 6 Departamento de Medicina Veterinária, Instituto Federal da Paraíba (IFPB), Avenida Presidente Tancredo Neves s/n, Sousa, PB 42800-605 Brazil

**Keywords:** Anaplasmosis, Babesiosis, Enzootic, Immunity, *Rhipicephalus microplus*, Tropic

## Abstract

We studied the immune condition of adult cattle to *Anaplasma marginale*, *Babesia bovis* and *Babesia bigemina* and identified characteristics influencing the transmission of tick fever (TF) agents in properties from the Recôncavo baiano, a region located in the Atlantic Forest biome in Brazil. Blood was sampled from cattle on 28 properties to determine the seroprevalence of antibodies against *A. marginale*, *B. bovis* and *B. bigemina* and define the inoculation rate of *B. bovis* and *B. bigemina* on each property. A correlation test was conducted between herd seroprevalences of the TF agents to search for common transmission routes. Relation between characteristics of each property (cattle information, handling practices, tick control practices and acaricide resistance) and *A. marginale* herd seroprevalence and *B. bovis* and *B. bigemina* herd infection rate were explored through comparative analyses. We found seroprevalences over 75% for the three TF agents, being a positive correlation between *A. marginale* and *B. bovis*. Variables influencing the transmission of TF agents were genetic composition, use of spray acaricide formulations, use of avermectins and use of fipronil. The present study shows that adult cattle from properties in the Recôncavo baiano region have high seroprevalences for the TF agents meaning that most of the adult cattle are immune and TF outbreaks should not be frequent. We suggest that the principal propagation route of *A. marginale* in properties from the Recôncavo baiano is tick-borne transmission. Further investigations are necessary to establish risk factors influencing the immunity situation of cattle in the region.

**Supplementary Information:**

The online version contains supplementary material available at https://doi.org/10.1007/s11250-026-04866-5.

## Introduction

The rickettsia *Anaplasma marginale* and the protozoans *Babesia bigemina* and *Babesia bovis* cause tick fever (TF), a complex of cattle diseases characterized by anorexia, somnolence, weakness, dry feces, tachypnea, fever, hemolytic anemia, and jaundice, which are followed by death if not treated (Richey 1992; Bock et al. 2004). The principal transmission route of the TF agents is their biologic vector, the cattle tick *Rhipicephalus (Boophilus) microplus*. Additionally, *A. marginale* can be transmitted mechanically by horseflies, *Stomoxys* spp. and/or blood-contaminated fomites as needles (Kocan et al. 2010). This clinical syndrome causes an important financial impact contributing substantially to the total economic loss caused by *R. microplus* in the Brazilian cattle herd. Beef cattle in the Brazilian Cerrado could lose until US$34.61 and US$7.97/animal in the backgrounding and finishing phase, respectively (Calvano et al. 2019).

In Brazil, the seroprevalence and inoculation rates of the TF agents vary between regions and properties due to differences in climatic and management practices, which influence the vector biology and their transmission. It has been considered that in regions where the climatic conditions favor tick development (Cerrado, Amazon and Atlantic Forest biomes) the constant transmission of TF agents will be enough to maintain herd immunity, and TF outbreaks would not be expected. In contrast, in regions where environmental conditions interfere with the development of *R. microplus* (chilly winter in the southern region, floods in the Pantanal, and low humidity in the Caatinga) the transmission of TF agents is limited to specific times of the year (Puentes and Riet-Correa 2023). Regarding management practices, the introduction of *Bos taurus* genotypes in farms to improve productivity, the employment of acaricide spraying and tick control malpractices have been factors influencing in the *A. marginale*,* B. bovis* and *B. bigemina*. seroprevalences (D’Andrea et al. 2006; Jonsson et al. 2008; Costa et al. 2013).

The climatic conditions in the Recôncavo baiano region favor *R. microplus* development throughout the year, so, it is possible that most of the cattle in this area have immunity against TF agents; however, the disease has been diagnosed occasionally causing relevant losses (Puentes et al. 2023). Given that there is no knowledge about immunity and/or conditions that influence the transmission of TF agents in cattle, we developed this research to investigate the immune condition of adult cattle to *A. marginale*, *B. bovis* and *B. bigemina* and to identify characteristics playing a role in the transmission risk of these agents in properties from the Recôncavo baiano region.

## Materials and methods

### Studied region

The Recôncavo baiano is part of the administrative division of the State of Bahia, Brazil. It has an extension of 4614 Km2 divided in 19 municipalities that share cultural and commercial relations: Cabaceiras do Paraguaçu; Conceição do Almeida; São Felipe; Cruz das Almas; Sapeaçu; Governador Mangabeira; Muritiba; Cachoeira; Maragogipe; Santo Amaro; Saubara; São Félix; Salinas da Margarida; Dom Macedo Costa; Nazaré; Santo Antônio de Jesus; Castro Alves; Muniz Ferreira; and Varzedo. The territory is in the Atlantic Forest biome, has a tropical humid climate and is characterized by well-defined seasons, a rainy winter from April to August, and a sunny summer from September to March. In 2024, the average temperature was 26.65 °C (± 4.43), the average relative humidity was 91.45% (± 12.77) and accumulated precipitation was 1148 mm. The Recôncavo baiano cattle herd is estimated to comprise 215 100 heads, corresponding to 1.7% of the bovine population in the State of Bahia (SEI 2024). The predominant cattle genotype is *Bos indicus* (94%), followed by *Bos taurus* (6%). In this territory, 79.7% of producers are small producers who practice subsistence production (IBGE 2017).

### Experimental design

**Fig. 1:**
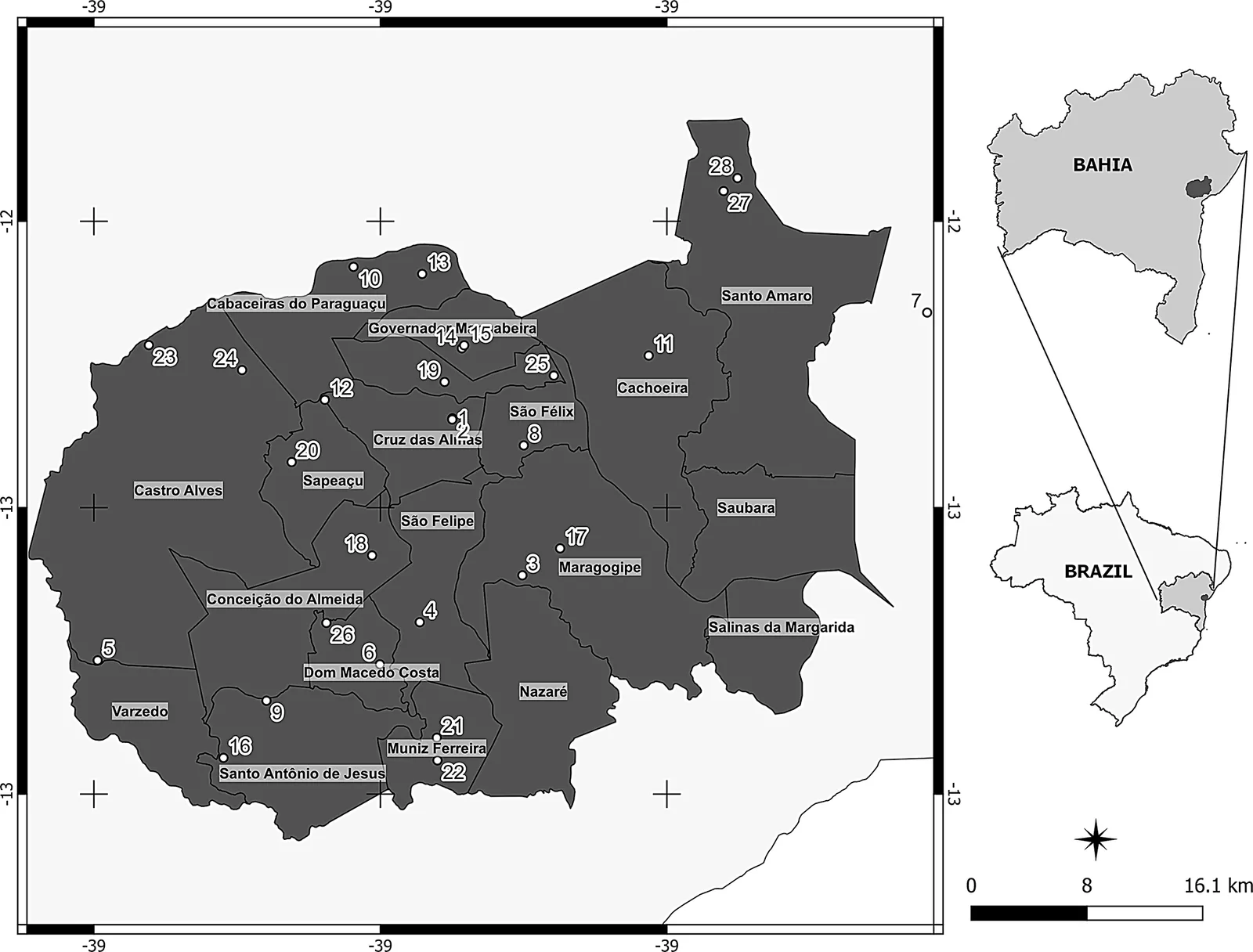
Localization of the sampled properties. The map shows the 19 municipalities that integrate the Recôncavo baiano territory. The yellow frame indicates the localization of the Recôncavo baiano territory and red points, the localization of the 28 sampled properties

We performed a cross-sectional analytic study. The sampling unit were properties. Between March and November 2024, we sampled by convenience 28 rural properties in the Recôncavo region of Bahia (Fig. 1). We made an especial effort to include properties from almost all the municipalities of the region. In the properties sampled, the average number of adult bovines were 34 (from 5 to 87 animals) and the average area, 61 ha. All properties produced cattle in extensive systems, 57% focused on beef and 43% on dairy production. 64% of the properties raised animals of *B. indicus* genotypes. All properties had animals infested with *R. microplus* and implemented acaricide control programs.

Given that adult bovines have been considered as the susceptible age group to TF and to prevent maternal immunity from interfering with the tests, we sampled male and female cattle between 9 months and 4 years of age, the same age range used in other reports to define enzootic scenarios of the disease (Mahoney and Ross 1972). The sample size in each property was calculated using the statistic software EpiInfo^®^ 7.2.4.0. version, allowing an acceptable error margin of 5%, a confidence level of 95%, and an expected frequency of 96% (Araújo et al. 1997, 1998). Once defined the size sample, we selected the animals using a simple random sampling method. We sampled a total of 554 cattle.

### Questionnaire

Information was obtained from each property owner or manager to characterize and identify factors influencing the *A. marginale* seroprevalence and the herd inoculation rate (*h*) of *B. bovis* and *B. bigemina.* The data collected from 18 questions included details about cattle information (herd size, genetic composition, production system), handling practices (cattle purchase) and tick and tick control practices (tick seasonality, frequency, criteria and methods for acaricide application, and acaricide compounds used).

### Blood sampling

The animals were restrained using cattle crushes or ropes. We collected the blood from the sacrocaudal vein in 10 mL tubes with clot activator. The tubes were centrifuged at 5000 rpm during five minutes. The serum was separated into 2 mL centrifugation microtubes of Eppendorf type and were frozen at -20 °C until being processed. The procedures were approved by the local committee on animal experimentation (CEUA-UFRB Nº 55/2023).

### Serology

To determine antibodies against TF agents in the sample, we used an indirect ELISA method described formerly (Machado et al. 1997; Ruiz et al. 2001). We employed solid plates with flat bottom Nunc-Immuno™ MicroWell™ (Sigma-Aldrich^®^, St. Louis, Missori). The antigens used were the 19 kilodaltons (kDa) recombinant major surface protein 5 (MSP5) of *A. marginale* (Visser et al. 1992), the 60 kDa recombinant roptria associated protein 1 (RAP1) of *B. bovis* (Suarez et al. 1991, 1993) and the 7 kDa clon C1A-GST, a fragment of the p200 antigen of *B. bigemina* (Tebele et al. 2000). The serum dilutions were 1:100 and 1:200 to *A. marginale* and *B. bovis* - *B. bigemina* diagnostic, respectively. The conjugated used was an Anti-Bovine IgG (whole molecule)-Alkaline Phosphatase produced in rabbit (Sigma-Aldrich^®^). The substrate employed was alkaline phosphatase 4-Nitrophenyl phosphate disodium salt hexahydrate (Sigma-Aldrich^®^), diluted in diethanolamine buffer solution with pH 9.6 (Sigma-Aldrich^®^). The intensity of the reaction was determined by a microplate reader with a 405 nm filter.

The cut-off point to define if the sample was positive was established by multiplying the mean optical density (OD) of the negative controls by 2.5, a constant / correction factor (Ruiz et al. 2001). We defined the cut-off points for *A. marginale*,* B. bovis* and *B. bigemina* seropositivity as 0.302, 0.307 and 0.335, respectively. The test was considered valid if the OD of the white well was < 0.090, the control positive, > 0.700 and the negative controls, between 0.100 and 0.200. The ELISA test for *A. marginale* has 94% specificity and 100% sensitivity; for *B. bovis*, 98.1% specificity and 98% sensitivity; and for *B. bigemia*, 94% specificity and 87.5% sensitivity (Madruga et al. 2000a, b; Ruiz et al. 2001).

### Herd inoculation rate (h) of *B. bovis* and *B. bigemina*

To determine the proportion of animals on each property that receive one infective inoculum of *B. bovis* and *B. bigemina* in one day, we defined the *h*. The *h* was calculated using the following formula: h = Ln (1 – I) / 270 – t; where Ln is the natural logarithm, *I* is the true herd seroprevalence of antibodies against *B. bovis* and *B. bigemina* and *t* is the average age in days of the sampled animals (we used 720 days for all the properties) (Mahoney and Ross 1972; Tice et al. 1998).

### Acaricide resistance bioassays

To evaluate if tick acaricide resistance may influence in the seroprevalence / inoculation rate of the TF agents, bioassays for five classes of acaricides were performed on ticks from seven properties. For cypermethrin (Sigma-Aldrich^®^) and chlorpyriphos (Sigma-Aldrich^®^) was performed the Larval Packet Test (LPT) (FAO 2004). For ivermectin (Sigma-Aldrich^®^) and fipronil (BASF Chemicals^®^, Paulínia, São Paulo, Brazil) was performed the larval immersion test (LIT) (Klafke et al. 2012; Castro Janer et al. 2015). For amitraz (Triatox^®^, MSD Saúde Animal, São Paulo, Brazil) was performed a modified LPT(Miller et al. 2002). The resistance ratio (RR) was calculated as the comparative susceptibility of the field population to a susceptible reference strain. Ticks were considered resistant when RR was > 2.0.

## Statistic analysis

First, we described the frequencies of seroprevalences of antibodies against *A. marginale*, *B. bovis* and *B. bigemina* at animal and herd level. At animal level, we calculated the percentage of all seropositive animals to *A. marginale*, *B. bovis* and *B. bigemina* with 95% confidence interval (CI) including continuity correction using the VassarStats software available in http://www.vassarstats.net/prop1.html. At herd level, we calculated the medians and interquartile ranges for the seroprevalences of antibodies against *A. marginale*,* B. bovis* and *B. bigemina*, and for the *h* of *B. bovis* and *B. bigemina*.

To explore common transmission route of *A. marginale*,* B. bovis* and *B. bigemina*, we looked for correlation between the herd seroprevalences of antibodies against those agents. We linearized the data through a square root transformation, then performed the Pearson test and executed simple lineal regression to establish if it could be an influence between variables. We just considered as valid those correlations with P value ≤ 0.05 and determination coefficient R2 ≥ 0.60.

To establish if the variables derived from the questionnaire influenced the herd seroprevalence of antibodies against *A. marginale* distribution and the *h* of *B. bovis* and *B. bigemina* distribution and Given that the dependent variables were continuous with non-free distribution, we compared the medians of both categories for each independent variable using the non-parametric Mann Whitney and Pearsson`s correlation test for continuous and numeric independent variables, respectively. The analyses were performed in the software The jamovi project (2025), *Jamovi* (Version 2.6) [Computer Software]. Retrieved from https://www.jamovi.org. The graphs were performed in the program Graph pad prism version 8.0.2 for Windows, GraphPad Software, Boston, Massachusetts USA, www.graphpad.com.

## Results

Regarding the information obtained through the questionnaire, the mean herd size of the properties sampled was 33.8 (CI: 25.6–41.9). Most properties are destined for beef production (57.15%, 16/28) and the rest, for dairy (45.85%, 12/28). Most of the herds were composed by Indian breeds (64.28%, 18/28), followed by properties with European breeds (35.72%, 10/28). Concerning cattle purchases, seven producers (25%, 7/14) reported purchasing cattle from other properties located in regions where climatic conditions favor the development of cattle ticks, such as the Atlantic Forest biome. Another seven (25%, 7/14) purchased cattle in regions where climatic conditions do not favor the parasite, such as the Caatinga biome. Relating to *R. microplus* infestation, most of the producers stated that the tick was present all through the year (64.28%, 18/28) and the remaining properties, that the infestation occured seasonally (35.72%, 10/28).

All producers notified that they employed chemical acaricides for tick control. Concerning the frequency of treatments per year, we found that it is significatively higher in properties with European than Indian breeds (*P* < 0.005), in dairy than in beef production systems (*P* < 0.05), in properties that did not perform injectable/avermectin acaricides (*P* < 0.05) and in properties that performed pour-on/fipronil formulations (*P* < 0.05) (Table S1). Most producers reported that applied acaricide only when high tick infestation is observed in cattle (67.86%, 19/28), while the rest treated in a systematic or prophylactic way (32.14%, 9/28). Most producers performed injectable (78.57%, 22/28) and pour-on acaricide formulations (78.57%, 22/28) and 67.87% (19/28) reported to use spray formulations. The most used acaricide compounds were pyrethroids (92.59%, 25/27), followed by organophosphates (85.18%, 23/27), avermectins (81.48%, 22/27), fipronil (48.14%, 13/27) and the less used is amitraz (3.70%, 1/27). Most producers did not use multiple acaricides classes simultaneously (57.14%, 16/28). The majority used different products with the same chemical class (53.57%, 15/27). Most producers did not alternate the acaricide compounds (60.71%, 17/27).

At animal level, Table 1 shows the frequencies of seropositive individuals to each one of the TF agents: 10.29% (CI: 7.95–13.2%) of the sampled animals were seropositive only for one agent (57/554), 14.44% (CI: 11.68–17.71%) for two agents (80/554), 69.67% (CI: 65.64–73.45%) for all three agents (386/554) and 5.6% (CI: 3.9–7.94%) were negative to the three agents (31/554). Regarding the animals seropositive for two agents, 20% (CI: 12.2-30.74%) were positive for *B. bovis* and *B. bigemina* (16/80), 33.75% (CI: 23.79–45.28%) for *A. marginale* and *B. bigemina* (27/80) and 46.25% (CI: 35.16–57.7%) for *A. marginale* and *B. bovis* (37/80). In all sampled properties, at least one animal seropositive for *A. marginale*,* B. bovis* and/or *B. bigemina* was detected.

**Table 1:** Frequencies of the seropositive individuals to each one of the TF agents

TF agent	Number of seropositive animals	Seropositive animals (%) (CI)
*Anaplasma marginale*	481/554	86.68 (83.65–89.47)
*Babesia bovis*	445/554	80.32 (76.71–83.5)
*Babesia bigemina*	449/554	81.05 (77.48–84.18)

Note: confidence interval, CI

**Fig. 2:**
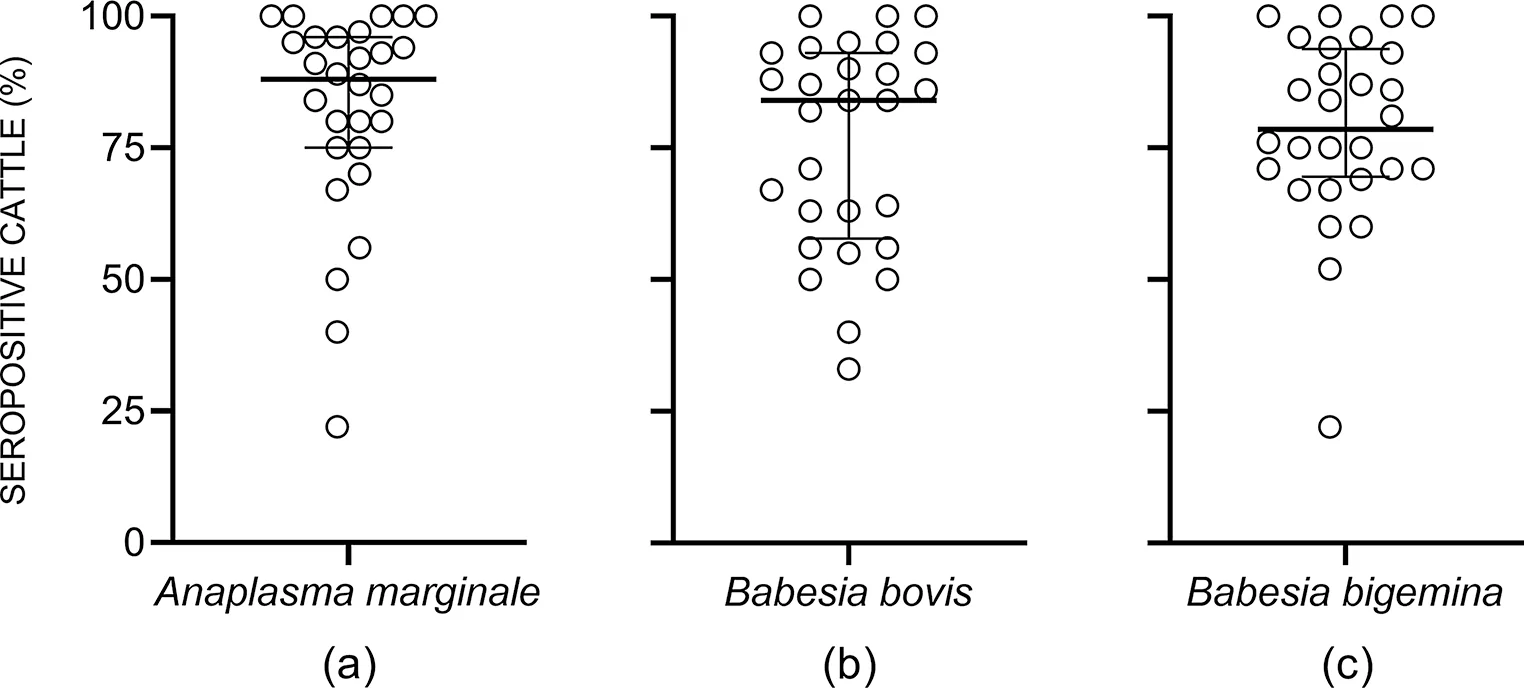
Herd seroprevalence of antibodies against *A. marginale* (**a**), *B. bovis* (**b**) and *B. bigemina* (**c**) in bovines between 9 months and 4 years of age. The results are presented as the medians (horizontal lines) and their interquartile ranges (error bars). Circles represent properties (*n* = 28)

**Fig. 3:**
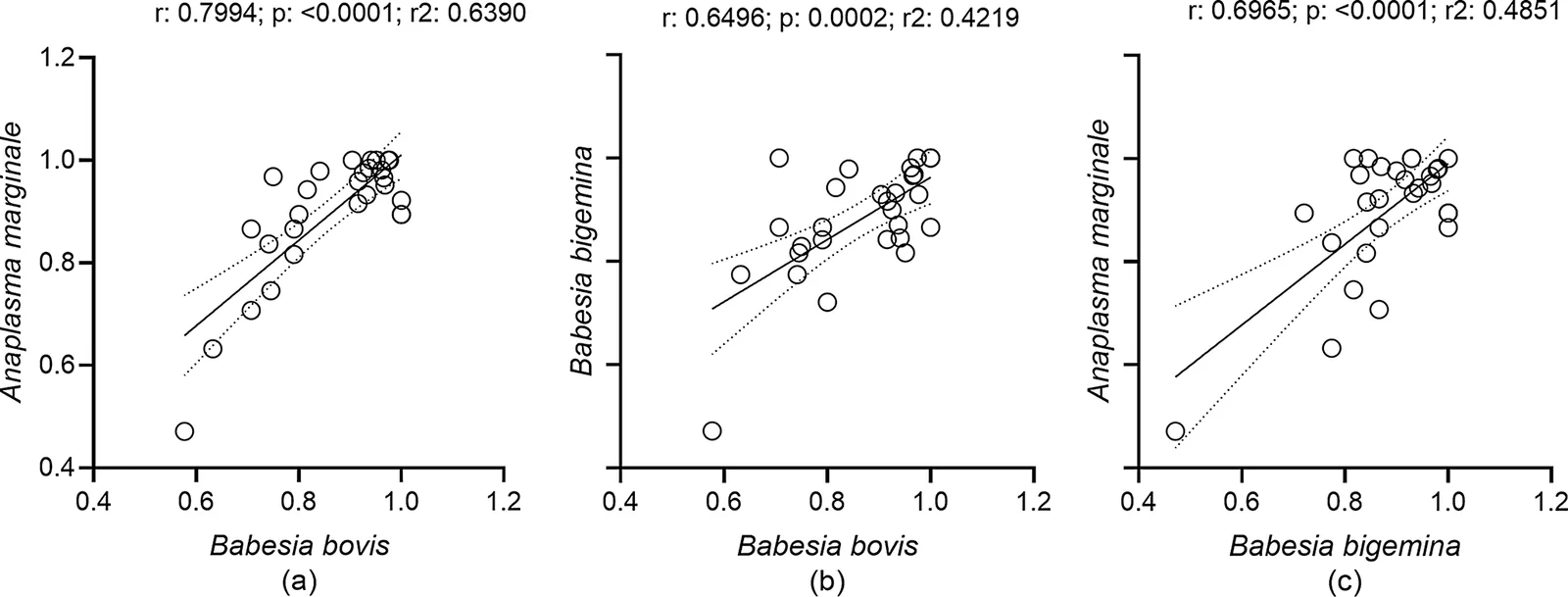
Correlation between the herd seroprevalence of antibodies against the TF agents. (**a**) Correlation between *A. marginale* and *B. bovis* herd seroprevalences. (**b**) Correlation between *B. bigemina* and *B. bovis* herd seroprevalences. (**c**) Correlation between *A. marginale* and *B. bigemina* herd seroprevalences. The percentage values were square root transformed. The correlation was performed through the Pearson test. Straight line represents the simple linear regression. Error bars (dashed lines) indicate the confidence intervals. Circles represent properties (*n* = 28)

At herd level, we sampled between 5 and 35 (mean:19.79; CI: 16.65–22.92) animals by property. The median seroprevalences of antibodies against *A. marginale*,* B. bovis* and *B. bigemina* were 88% (IQR:75–96%), 84% (IQR:57.75-93%) and 78.5% (IQR:69.5-93.75%), respectively (Fig. 2). A strong correlation was determined between the herd seroprevalences of antibodies against *B. bovis* and *A. marginale* (r: 0.7994; *P*: <0.0001; r2: 0.6390) demonstrating that 63.9% of the *B. bovis* herd seroprevalence variance is explained because of the *A. marginale* herd seroprevalence (Fig. 3a). We did not find a correlation between *A. marginale* and *B. bigemina*, nor between *B. bovis* and *B. bigemina* herd seroprevalences either (Fig. 3b, c). Regarding the proportion of animals on each property that receive one infective inoculum of *B. bovis* or *B. bigemina* in one day, the *h* medians were 0.0034 (IQR:0.0026–0.0061) and 0.0041 (IQR:0.0019–0.0059) for *B. bigemina* and *B. bovis*, respectively.

Table 2 describes the comparation between medians for each independent variable from the questionnaire. The *A. marginale* seroprevalence median was significatively lower in properties where acaricide injectable formulations (*P* < 0.05), avermectins (*P* < 0.05) or fipronil (*P* < 0.05) are employed. The *B. bovis h* median was significatively higher in properties with Indian breeds (*P* < 0.05), where acaricide injectable formulations (*P* < 0.005) and avermectins are used (*P* < 0.005) and was significatively lower in properties where acaricide spray formulations are avoided (*P* < 0.05). The *B. bigemina h* median was significatively higher in properties with Indian breeds (*P* < 0.05) and in properties where spray acaricide formulations are performed (*P* < 0.05). No correlation was found between herd size and the seroprevalence / inoculation rate of the TF agents (Table S2).

**Fig. 4:**
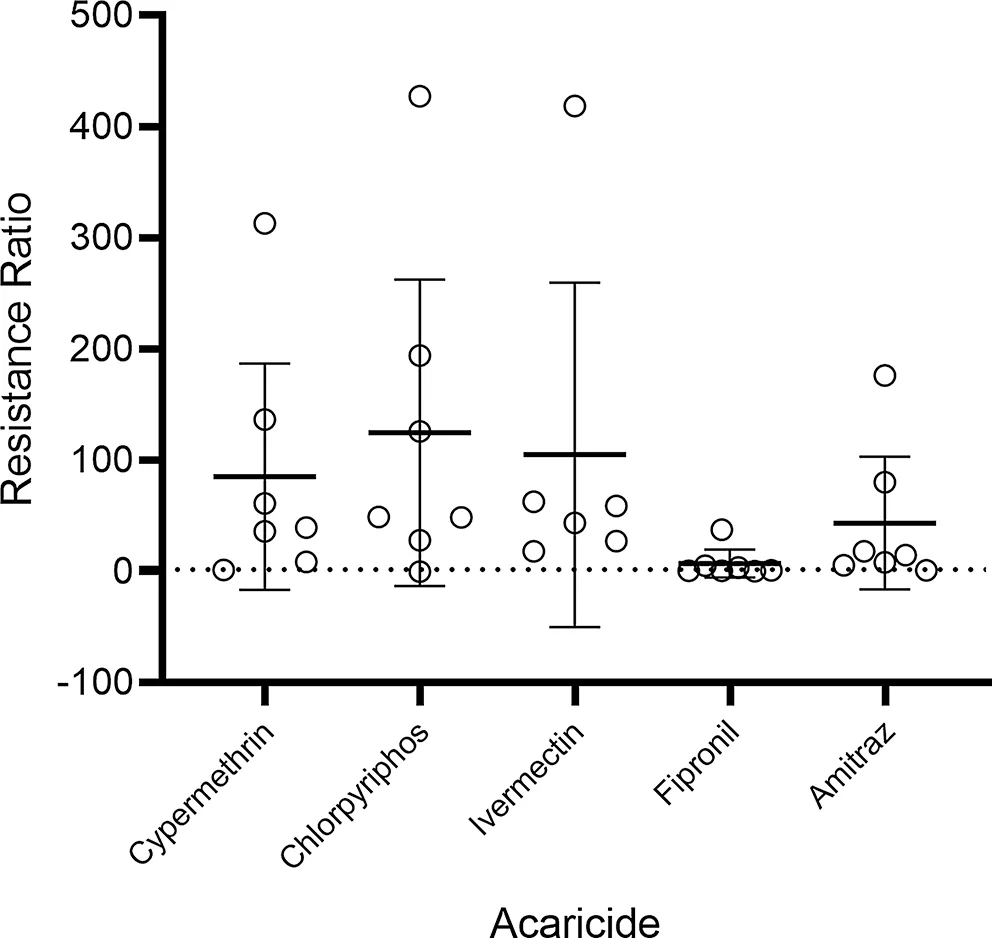
Distribution of resistance ratios of *Rhipicephalus microplus* populations from seven properties in the Recôncavo baiano region, State of Bahia, Brazil. The results are presented as the means (horizontal lines) and the confidence interval (error bars). The dashed line indicates the RR threshold (2.0) from which ticks would be considered resistant. Circles represent properties (*n* = 7)

Regarding the acaricide resistance bioassays performed on seven properties, the mean RR for cypermethrin was 85.40 (CI: -16.27;187.1), and susceptibility was observed in only one property; for chlorpyrifos, 124.8 (CI: -12.81;262.5), and susceptibility was observed in only one property; for ivermectin, 105 (CI: -57.37;267.4) and resistance occurred in all properties; for fipronil, 7.16 (CI: -5.35;19.67) and susceptibility was observed in four properties; and for amitraz, 43.59 (CI: -16.07;103.2) and susceptibility was observed in only one property (Fig. 4).

**Table 2:** Medians of the herd *A. marginale* seroprevalence, *B. Bovis* and *B. bigemina*. *h* distribution compared across 18 categoric factors of 28 properties from the Reconcavo Baiano region, State of Bahia, Brazil

	*A. marginale* seroprevalence			*B. bovis* h			*B. bigemina* h		
**Independent variable (n)** Category (n)	**Median** **(%)**	**IQR** **(%)**	**p**	**Median** **(h)**	**IQR** **(%)**	**p**	**Median** **(h)**	**IQR** **(%)**	**p**
**Genetic composition (28)**			0.229			**0.004**			**0.023**
European (10)	22.5	9.75–25.8		0.002	0.002–0.002		0.003	0.002–0.003	
Indian (18)	14	4-19.8		0.005	0.004-0.00675		0.004	0.003-0.00675	
**Production system (28)**			0.149			0.064			0.194
Beef (16)	14	3.5–19.3		0.0045	0.00375–0.00625		0.004	0.003-0.00625	
Dairy (12)	22.5	5.5–26.3		0.002	0.002-0.00525		0.003	0.002-0.00525	
**Cattle reposition (14)**			0.44			0.948			0.218
From Atlantic Forest biome (7)	18	5-24.5		0.005	0.003–0.008		0.004	0.003–0.006	
From Caatinga biome (7)	6	3–18		0.006	0.0025–0.007		0.007	0.005–0.01	
**Tick infestation occurrence (28)**			0.336			0.624			0.542
Part of the year (10)	20.5	16.8–23.5		0.0045	0.0025–0.00575		0.0035	0.003-0.00575	
All through the year (18)	11.5	4-24.5		0.0035	0.002-0.00575		0.0035	0.002–0.0065	
**Criteria for acaricide treatments (28)**			0.361			0.466			0.367
Systematic or prophylactic (9)	18	4–20		0.004	0.002–0.007		0.004	0.003–0.006	
High tick infestation in cattle (19)	16	5-26.5		0.004	0.002–0.005		0.003	0.0025–0.0055	
**Injectable acaricide formulations (28)**			**0.026**			**0.002**			0.082
Yes (22)	11.5	4-19.8		0.005	0.00325-0.006		0.004	0.003-0.00675	
No (6)	24	22.3–25.8		0.002	0.00125-0.002		0.003	0.00225-0.003	
**Pour-on acaricide formulations (28)**			1.000			0.529			0.776
Yes (22)	17	4.5–23.8		0.004	0.002–0.006		0.0035	0.003-0.00575	
No (6)	17.5	7-24.3		0.003	0.002-0.00475		0.003	0.002–0.0085	
**Spray acaricide formulations (28)**			0.554			**0.029**			**0.048**
Yes (19)	18	4-25.5		0.002	0.002–0.005		0.006	0.004–0.007	
No (9)	11	6–20		0.006	0.004–0.006		0.003	0.002–0.0045	
**Use of avermectins (ivermectin - doramectin) (27)**			**0.026**			**0.003**			0.080
Yes (22)	11.5	4-19.8		0.005	0.00325-0.006		0.004	0.003-0.00675	
No (5)	25	22–26		0.002	0.001–0.002		0.003	0.002–0.003	
**Use of pyrethroids (27)**			0.676			0.572			0.480
Yes (25)	16	4–24		0.004	0.002–0.006		0.003	0.003–0.006	
No (2)	19.5	19.3–19.8		0.005	0.0045–0.0055		0.005	0.0045–0.0055	
**Use of amitraz (27)**			0.334			0.360			0.601
Yes (1)	25	25–25		0.002	0.002–0.002		0.005	0.005–0.005	
No (26)	16	4-22.8		0.004	0.002–0.006		0.0035	0.003–0.006	
**Use of fipronil (27)**			**0.036**			0.152			0.374
Yes (13)	6	2–20		0.005	0.002–0.007		0.004	0.003–0.006	
No (14)	21	12.3–27.5		0.0035	0.002–0.005		0.003	0.00225–0.00575	
**Use of organophosphates (27)**			0.732			0.466			0.972
Yes (23)	12	4-24.5		0.004	0.002–0.0055		0.004	0.003–0.0065	
No (4)	18.5	17.5–19.3		0.005	0.0035–0.007		0.0035	0.003–0.0045	
**Rotation of acaricide compound (27)**			0.579			0.459			0.818
Yes (10)	14	2.5–23.3		0.004	0.002-0.00475		0.0035	0.003-0.00475	
No (17)	18	6–23		0.005	0.002–0.006		0.004	0.003–0.007	
**Multiple acaricides classes used simultaneously (28)**			0.235			0.218			0.237
Yes (12)	16	3.5–20.3		0.0045	0.002–0.007		0.004	0.003–0.007	
No (16)	20.5	6-26.3		0.0035	0.002–0.005		0.003	0.002-0.00525	
**Use different products with same compound (27)**			0.607			0.487			0.551
Yes (15)	16	4-24.5		0.004	0.002–0.005		0.003	0.003–0.005	
No (12)	14.5	5.5–20.5		0.0045	0.00275–0.00625		0.004	0.003–0.007	

Note: Mann Whitney test. Variables with *P* values in bold were considered significative. Interquartile range, IQR. Herd inoculation rate, *h*

## Discussion

In the current research, we analyzed the immune state of adult bovines against *A. marginale*, *B. bovis* and *B. bigemina* and identified characteristics that influence the transmission of these agents in herds from the Recôncavo baiano region. Our results demonstrate that the TF agents seroprevalences are high in cattle properties from the region. Also, the cattle tick, *R. microplus*, plays an important role in the *A. marginale* transmission. After an infection with *A. marginale*, cattle have lifelong immunity, while antibodies against *B. bovis* last at least four years and antibodies against *B. bigemina* generally persist for less than six months (Mahoney et al. 1973; Kocan et al. 2010). Therefore, developing research based on immunological studies against TF in adult cattle provides valid information on the epidemiological aspects of this disease.

The fact that 75% of the properties sampled had herd seroprevalences over 75%, 57.75% and 69.5% to *A. marginale B. bovis* and *B. bigemina*, respectively, suggest that most of the cattle have immune protection and, although the agents circulate in the region, outbreaks of TF would be uncommon. In the Recôncavo baiano, *R. microplus* is present the entire year, so cattle persistently exposed to infection are producing protective antibodies continuously (concomitant immunity) (Brown et al. 2006). High herd seroprevalences against *A. marginale*,* B. bovis* and *B. bigemina* have been found in adult cattle from other regions of Brazil (Guedes Junior et al. 2008; Souza et al. 2013; Costa et al. 2015). Although several works around the world have attempted to understand and predict the TF risk through the concept of enzootic condition based in works from regions with ecological and host genotype differences, it would not be adequate to replicate in the present work. To define the TF enzootic situation in the Recôncavo baiano region it would be necessary to apply a prospective study to stablish the *h* ranges of *A. marginale*,* B. bovis* and *B. bigemina* in both adults and calves, where it would not be expected TF outbreaks (enzootic stability) and where it would be the maximum TF outbreak risk (enzootic instability) confirming the cases through an accurate diagnosis (Jonsson et al. 2012).

In the Recôncavo baiano region, it seems that the major transmission route of *A. marginale* is the intraestadial transmission by *R. microplus* because of the positive correlation found between *A. marginale* and *B. bovis* herd seroprevalences. Other authors also have made this assumption given that *R. microplus* is the exclusive vector of *B. bovis* (Ramos et al. 2020; Morel et al. 2024). This premise make sense given that, in enzootic stable zones, the cattle tick is responsible for the endemic situation of anaplasmosis. (Kessler 2001). Besides, the mechanical transmission seems to have little relevance because persistent infected animals from enzootic stable zones just get rickettsemias between 10^2.5^ and 10^6^ infected erythrocytes (IE)/mL of blood, so, it is necessary rickettsemias above 5 × 10^6^ IE/mL to horseflies transmit the minimal infectious dose (Scoles et al. 2008). It has not been demonstrated transmission trough blood-contaminated fomites from animals with rickettsemias under 1.45 × 10^8^ IE/mL (Reinbold et al. 2010).

The lower *h* of *B. bovis* and *B. bigemina* in properties with European breeds could be related to the higher frequency of acaricide applications compared to properties with Indian breeds. This is plausible given that *R. microplus*, the only vector of *B. bovis* and *B. bigemina* in cattle, is present throughout the year in our studied region and most of the sampled properties do not receive technical assistance in implementing their tick management strategies, which could result in acaricide misuse and different levels of infestation across properties (Klafke et al. 2024). Another possible reason could be that the animals we sampled would never have been exposed to tick; however, all properties raise animals in contact with *R. microplus* and none of the *B. bovis* or *B. bigemina h* were associated with the recent introduction of cattle from tick-free regions.

Properties using acaricide spray formulations had lower *B. bovis* transmission but higher *B. bigemina* transmission. This could be explained based on the evolutive stages of transmission of these protozoans. Given that *B. bovis* is transmitted by the larval stage of *R. microplus*, while *B. bigemina* is transmitted by the nymphal stage, properties using spray formulations could impact more on larvae than nymph or adult tick stages, interfering with *B. bovis h* (Esteve-Gasent et al. 2020). Tick larvae are more susceptible to contact acaricides because its cuticle is far less tough than the hard adult providing better penetration of the product (Gaur et al. 2016).

The increased transmission of *B. bovis* but decreased transmission of *A. marginale* on properties using avermectins (ivermectin - doramectin) for tick control is possible because avermectins had no effect on *R. microplus* larvae (the *B. bovis* vector), as observed in the ivermectin bioassay. However, because this compound has a long residual period, it may affect adult male ticks, the rickettsia vector (Davey et al. 2010). Properties using fipronil had lower seroprevalence of *A. marginale* probably because this acaricide was effective against ticks in most properties, limiting *A. marginale* transmission. Although we attempted to establish the impact of tick acaricide resistance on the TF agent’s transmission, this would require bioassays to be performed on all properties.

The major difficulty in this research was that we had to choose properties interested in participating by convenience (non-randomized sampling). Although our results could not be extrapolated to all the Recôncavo baiano region, they could reflect in the reality of the region and represent a contribution to the understanding of the *A. marginale*,* B. bovis* and *B. bigemina* immune condition in regions and properties with characteristics very similar to our sample (small and medium properties located in tropical regions from Atlantic forest near to semiarid biome). These kinds of regions are in Brazilian states as Bahia, Sergipe, Alagoas, Pernambuco, Paraiba and/or Rio Grande do Norte.

The present study shows that adult bovines from properties in the Recôncavo baiano region have seroprevalences for the TF agents over 75%, meaning that most of the adult cattle are immune and TF outbreaks should not be frequent. Although factors like genetic composition, class of acaricide compound and formulation used were indirectly associated with variations in the transmission of TF agents, further investigations are necessary to establish risk factors influencing the immunity situation of cattle in the region. 

## Supplementary Information

Below is the link to the electronic supplementary material.


Supplementary Material 1


## Data Availability

All data generated in this study are included in this published article.
