# Supplementary material for: Tick fever risk and factors influencing the transmission of Anaplasma marginale, Babesia bovis and Babesia bigemina in cattle properties from Atlantic Forest biome
Source: Trop Anim Health Prod. 2026 Jan 30;58(2):73. doi: 10.1007/s11250-026-04866-5 (PMC12858521; doi:10.1007/s11250-026-04866-5)
Supplement: Supplementary file 1 — Supplementary Material 1 [file 11250_2026_4866_MOESM1_ESM.docx]

**Table S1** Medians of acaricide application frequency per year compared across 18 categoric factors of 28 farms from the Reconcavo baiano region, State of Bahia, Brazil

|  | Acaricide frequency / year | | |
| --- | --- | --- | --- |
| **Independent variable (n)**  Category (n) | **Median** | **IQR** | **p** |
| **Genetic composition (28)** |  |  | **<0.001** |
| European (10) | 11 | 6-20.8 |  |
| Indian (18) | 2 | 2-4 |  |
| **Production system (28)** |  |  | **0.008** |
| Beef (16) | 2 | 2-4 |  |
| Dairy (12) | 8 | 5.25-18.3 |  |
| **Cattle reposition (14)** |  |  | 0.558 |
| From Atlantic Forest biome (7) | 4 | 2-11 |  |
| From Caatinga biome (7) | 4 | 2-5 |  |
| **Tick infestation occurrence (28)** |  |  | 0.281 |
| Part of the year (10) | 6 | 2.25-9 |  |
| All through the year (18) | 4 | 2-6 |  |
| **Acaricide purchase (28)** |  |  | 0.151 |
| Veterinary recommendation without resistance test (13) | 3 | 2-6 |  |
| Personal election (15) | 6 | 2-11 |  |
| **Criteria for acaricide treatments (28)** |  |  | 0.436 |
| Systematic or prophylactic (9) | 6 | 2-17 |  |
| High tick infestation in cattle (19) | 4 | 2-6 |  |
| **Injectable acaricide formulations (28)** |  |  | **0.013** |
| Yes (22) | 3.5 | 2-6 |  |
| No (6) | 17 | 7.5-23.5 |  |
| **Pour-on acaricide formulations (28)** |  |  | **0.03** |
| Yes (22) | 5 | 2.25-10 |  |
| No (6) | 2 | 2-2 |  |
| **Spray acaricide formulations (28)** |  |  | 0.303 |
| Yes (19) | 4 | 2-11 |  |
| No (9) | 3 | 2-6 |  |
| **Multiple acaricides classes** **used simultaneously (28)** |  |  | 0.924 |
| Yes (12) | 4 | 2-7 |  |
| No (16) | 4 | 2-7 |  |
| **Use of avermectins (27)** |  |  | **0.017** |
| Yes (22) | 3.5 | 2-6 |  |
| No (5) | 22 | 12-24 |  |
| **Use of pyrethroids (27)** |  |  | 0.85 |
| Yes (25) | 4 | 2-10 |  |
| No (2) | 4 | 3-5 |  |
| **Use of amitraz (27)** |  |  | 0.168 |
| Yes (1) | 22 | 22-22 |  |
| No (26) | 4 | 2-6 |  |
| **Use of fipronil (27)** |  |  | **0.016** |
| Yes (13) | 6 | 4-17 |  |
| No (14) | 2 | 2-3.75 |  |
| **Use of organophosphates (27)** |  |  | 0.917 |
| Yes (23) | 4 | 2-8 |  |
| No (4) | 4 | 2-7 |  |
| **Rotation of acaricide active principle (27)** |  |  | 0.061 |
| Yes (10) | 6 | 4-15.3 |  |
| No (17) | 2 | 2-6 |  |
| **Use different products with same active principle (27)** |  |  | 0.96 |
| Yes (15) | 4 | 2-8 |  |
| No (12) | 4 | 2-7 |  |

Note: Mann Whitney test. Variables with *P* values in bold were considered significative. IQR, interquartile range.

**Table S2** correlation between herd size and the seroprevalence / inoculation rate of the TF agents

|  | *A. marginale* seroprevalence | *B. bovis* *h* | *B. bigemina h* |
| --- | --- | --- | --- |
| **Pearson’s r** | 0.165 | -0.259 | -0.014 |
| **Df** | 26 | 26 | 26 |
| **P** | 0.403 | 0.183 | 0.945 |

Note: Pearson’s correlation test. Df: degrees of freedom
